# The Prevalence of the Burnout Syndrome and Factors Associated in the Students of Dentistry in Integral Clinic: A Cross-Sectional Study

**DOI:** 10.1155/2023/5576835

**Published:** 2023-08-21

**Authors:** Luis Alberto Chauca Bajaña, Luis Campos Lascano, Lourdes Jaramillo Castellon, Carlos Carpio Cevallos, Gabriela Cevallos-Pozo, Byron Velasquez Ron, Fábio França Vieira e Silva, Mario Perez-Sayans

**Affiliations:** ^1^Periodontics and Implantology Oral Research Unit, College Dentistry, University of Guayaquil, Guayaquil, Ecuador; ^2^Faculty of Dentistry, University of Guayaquil, Guayaquil, Ecuador; ^3^Salesian Polytechnic University, Quito, Ecuador; ^4^Dental Prosthesis Department Research, College Dentistry, University of the Americas (UDLA), Quito, Av. Colon y 6 de diciembre, Colón Campus, Quito, Ecuador; ^5^Faculty of Medicine and Dentistry, Oral Medicine, Oral Surgery and Implantology Unit, Health Research Institute of Santiago de Compostela (IDIS), University of Santiago of Compostela, Santiago of Compostela, A Coruña, Spain

## Abstract

**Background:**

Burnout syndrome (BS) is composed of three interrelated dimensions (emotional exhaustion, depersonalization, and personal fulfillment), and it has been documented that it affects health professionals from an early age.

**Aims:**

Determine the prevalence of BS and associated factors in the integral clinic of the Dentistry Pilot School. *Material and Methods*. Two instruments were applied: (1) Maslach Burnout Inventory, which measures the degree of professional burnout through 22 items that describe the professional's attitudes and feelings toward work, as well as symptoms associated with this phenomenon; (2) the second questionnaire determines the possible symptoms of BS and consists of 14 questions that describe tiredness, sleep problems, digestive problems, respiratory problems and headaches, temporomandibular joint (TMJ), neck pain, back pain, and upper and lower extremity pain. The instruments were answered anonymously by a total of 300 students who participated in the study.

**Results:**

The emotional exhaustion of the participants was 48.3% at a higher level, the depersonalization was 46.7% at a higher level, and the low perception of personal fulfillment was 73%. In addition, it was shown that BS is significantly related to marital status (*p* < 0.001^*∗*^), with single people reporting being more exhausted, with the 6-month level (*p* = 0.011) and with the following symptoms: non-neck pain, head, TMJ, back, waist, upper and lower body pain.

**Conclusion:**

It was found that the BS had a prevalence of high levels of exhaustion and depersonalization correlated with the marital status and level of preparation (academic degree) of the person, finding a prevalence of symptoms such as pain in the neck, head, TMJ, and back.

## 1. Introduction

Burnout syndrome (BS) was first described in 1974 by Freudenberger [1]. That syndrome is defined as an inadequate response to emotional distress and an excessive need to cope with chronic interpersonal stressors in the workplace [2, 3]. Malash describes it in three interrelated dimensions: emotional exhaustion, depersonalization, and lack of personal attendance. [4, 5] It is reported that BS affects between 20% and 50% of health professionals [6] and that the prevalence of BS among dentists is between 8% and 36% [7, 8]. Some authors have determined stressful moments in medical students, and it is considered that medical training has a high psychological toxicity [9]. Additionally, in a systematic review, they concluded that the COVID-19 pandemic has had a significant impact on people's mental health, stress, anxiety, and temporomandibular disorders (TMD), with headaches being common responses experienced [10]. Minervini et al. [11] described that TMD is an increasingly frequent problem in children and adolescents, and their prevalence varies between 20% and 60%. People that live with Gaucher disease due to various factors and symptoms, such as bone pain and fatigue, can limit a person's ability to cope, carry out daily activities, work, or enjoy daily life, and this can generate stress and frustration. Stress is not a direct cause of Gaucher disease but can play a role in the emotional and physical well-being of people with this condition [12]. In addition, dental students may have coping strategies that are classified as adaptive or maladaptive [13, 14]. Maladaptive coping has been described as worse academic performance and psychological outcomes in dental students [15]. Inadequate prevention and late arrest of BS can be evidenced throughout the entire career, which is why suicidal ideation, alcohol, and drug use increases [16]. That is the motive of the BS is recognized as a phenomenon that has negative impacts on academic training, being a predictor of worse quality of life [17]. Stress factors affect the academic training of university students since they lower their performance, increase emotional exhaustion, and the future professional life of students is damaged. Understand that the aim of this study is to determine the prevalence of BS and associated factors in the integral clinic of Dentistry Pilot School (University of Guayaquil).

## 2. Materials and Methods

**Figure 1:**
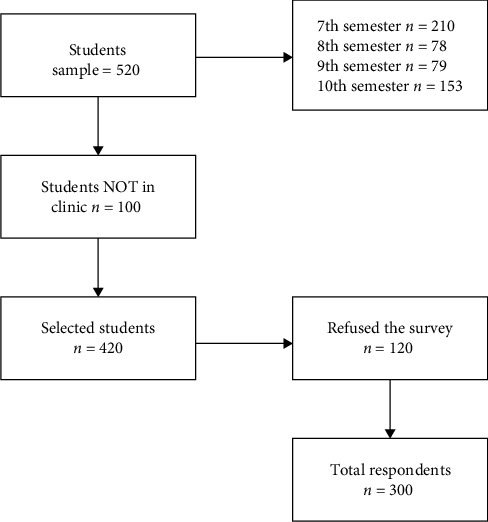
Flow diagram research.

An exploratory, analytical, correlational, and cross-sectional study is performed in this work. Students from the Dentistry Pilot School legally enrolled in a comprehensive clinic of the 7th, 8th, 9th, and 10th semesters of cycle *I* (2022–2023) are selected. A letter for authorization is sent to the Dean of the School of Dentistry, explaining the application of the data collection instrument to the students. This project has been approved by the Bioethics Committee of the University of Santiago de Compostela (Ethical Committee USC 47/2022). The application was carried out virtually through a link that reached the institutional email of the participants; teachers were asked to allow allocating a fraction of the time of their classes, facilitating their answer to the questionnaire. The data collection was carried out, over a period of 10 days, through the Google Forms application with relevant information about the study, implications, and informed consent. The total sample of 520 students legally enrolled, 100 students were absent from the clinic, and 120 did not agree to participate in the study; the sample was defined as 300 students who answered the survey (Figure 1).

**Figure 2:**
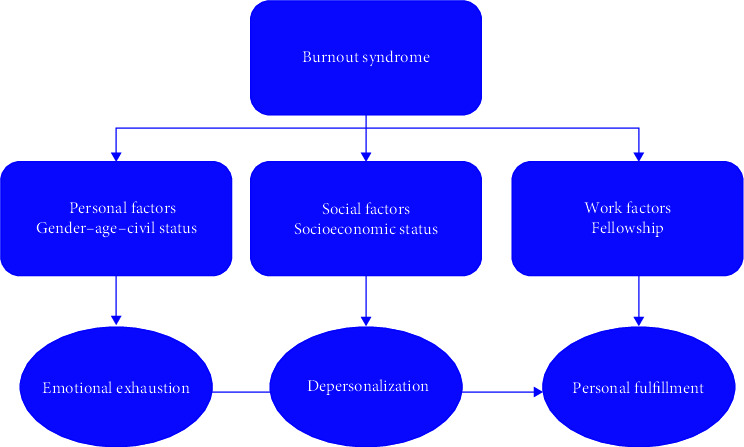
Burnout syndrome factors [4].

The participants anonymously answered two questionnaires, the first of the Maslach Burnout Inventory (MBI) [18], which is made up of 22 questions dealing with professional burnout to describe attitudes and feelings related to work. The three main dimensions measured by the MBI are exhaustion or emotional exhaustion (EE), depersonalization subscale (D), and personal fulfillment (PF). The subscales (EE, D, and PF) should be kept separate, and the scores on each subscale are ranked according to the percentile of each scale. Maslach allows professional BS to be classified into three groups according to the score obtained (no risk: 0–43 points; trend: 44–87 points; BS: >88 points). This questionnaire was validated by Schaufeli and by Carlotto in Brazil [19, 20] (Figure 2).

The second questionnaire determines the possible symptoms of BS and consists of 14 questions that describe tiredness, sleep problems, digestive problems, respiratory problems and headaches, temporomandibular joint (TMJ), neck pain, back pain, and upper and lower extremity pain [21, 22].

These questionnaires were used in a pilot study of random students (*n* = 30). The pilot study aimed at the perception of the questions in the students, and based on that, modified the questions for a better outcome in the investigation; also evaluated the time of completion of the questionnaire (approximately 10 min); there were no confusing questions and neither difficulty in compression, this was discussed with the group of students. In addition, they were evaluated by frequency using the Likert scale (0–1): 0 (never), 1 (almost never), 2 (sometimes), 3 (regularly), 4 (quite often), 5 (almost always), and 6 (always) [23].

## 3. Results

In this study, 300 students from the seventh to tenth semester participated; 71% of the sample was between 21 and 23 years of age. Of the participants, 94 (31.33%) were male, and 206 (68.67%) were female. The prevalence of BS is evident at low levels of personal fulfillment and at high levels of emotional exhaustion and depersonalization (Table 1). A descriptive analysis of BS analyzes emotional exhaustion, depersonalization, and personal fulfillment (Table 2).

On the distribution of symptoms of back and waist pain, tension, and neck pain, in descending order of frequency Table 3.

The results in sociodemographic, academic, and symptom characteristics were variable, as we can see in Table 4.

## 4. Discussion

### 4.1. General Discussion

Currently, BS is considered work-related stress that is characterized by generating emotional exhaustion, depersonalization, and feelings of low personal or professional accomplishment in those who experience the syndrome [24]. This study aimed to evaluate the prevalence of BS in students of a comprehensive dental clinic. The MBI for professionals was applied because the participants, in addition to being students, were carrying out their clinical work practices in dentistry. According to Jiménez-Ortiz et al. [24], professionals trained in dentistry have a greater tendency to experience BS than other health professionals due to the way they carry out their work and the large number of demands placed on them. Among the main results, a high prevalence of BS was found in the study population, reporting high levels of emotional exhaustion (48.3%), depersonalization (46.7%), and low levels of personal fulfillment (73%). This high level of occurrence requires professional training.

These results coincide with studies carried out in other countries, such as the study by Gorter et al. [25] in which high levels of burnout were found, especially in women, as well as in the study by Pereira et al. [26] it was found that dental students presented high levels of burnout, finding that personality factors play an important role when measuring burnout, since the trait of perfectionism can generate greater vulnerability in students to experience this phenomenon. On the other hand, our study was carried out in a Spanish sample in which 77.8% of the participants presented a medium risk of experiencing BS, as well as the association between this syndrome and the number of working hours and the environment [4].

The findings found in similar contexts on the levels of exhaustion reported by dental students provide an alarming perspective, so the use of coping strategies that can be incorporated by university institutions is imperative to avoid academic desertion [27], as well as also a low motivation of the student about his career and a bad interaction with his patients [28]. Additionally, regarding the relationship between the dimensions of the BS and the sociodemographic variables, in the present study, statistically significant relationships were found with the marital status variable, with singles reporting higher levels of burnout, possibly due to the fact that students since, they do not have family responsibilities such as children, they tend to overfocus on the professional demands that the career demands of them and dedicate more time to activities such as clinical practices and work. This finding is striking because the students who have been reported to be more exhausted are mostly those who are married or divorced, as is the study carried out by Al-Zain and Abdulsalam [29]. Likewise, it was also found that gender, age, and stress are significant predictors of emotional well-being (*p* < 0.0001), and exhaustion had a moderately significant positive relationship with stress and a weak negative relationship with resilience and determination conditions employment were significant [29].

In contrast, in academic variables, a statistically significant relationship between emotional exhaustion and the variable of the semester studied can be evidenced, with the 10th-grade students reporting the highest levels of exhaustion. Probably since 10th graders are finishing their academic work and are beginning to incorporate work and family obligations into their agenda, so they tend to feel more exhausted [27]. Thus, the university institution can have an idea of the strategies to be used at each level of the professional career, especially in those students who are studying the last semester to prevent a low sense of satisfaction with the career due to their levels of BS [30].

The present study focused on the symptoms presented by dental students, in which statistically significant relationships were found between BS and symptoms such as difficulty sleeping (*p* < 0.001), muscle tension (*p* < 0.001), not being able to enjoy activities (*p* < 0.001), headaches (*p* < 0.001), neck pain (*p* < 0.001), TMJ pain (*p* < 0.001), extremities pain (*p* < 0.001), back (*p* < 0.001), digestive discomfort (*p* < 0.001), loss of appetite (*p* < 0.001), nausea (*p* < 0.001), tremor in the hands and eyelids (*p* < 0.001), respiratory problems (*p* < 0.001) and frequent tiredness (*p* < 0.001), while symptoms such as increased appetite were significantly (*p* < 0.026) related to personal accomplishment.

In this case, these results coincide with the literature in reflecting the physiological repercussions that high levels of BS can generate in dental students. The study carried out by Metlaine et al. [31] in a sample of 1,300 workers reported a statistically significant relationship between high levels of burnout and sleep disorders such as insomnia in workers, 16.8% of the sample suffered from insomnia. The figures and symptoms evaluated reveal the great importance of the correct measurement of BS in students and workers for the use of coping strategies and work on stress management in order to minimize the impact that this phenomenon generates in the population studied [32].

### 4.2. Practice Implications

People who experience BS feel emotionally exhausted and worn out, leading to a loss of energy and motivation, which significantly impacts their professional, academic, and personal performance, reverberating in their physical, mental, and emotional health [33]. Dental students dedicate theoretical and practical academic hours that reach 12 hr a day, and this fact generates emotional and physical exhaustion [34]. The findings in the literature suggest educating students and faculty about the signs and symptoms of BS to prevent detrimental effects that can inhibit their academic success [35]. Using engagement strategies such as problem-solving, constructive reflection, and emotional expression could help students manage stress and burnout. All institutional measures for the distribution of hours and tasks must be humanized and directed toward expanding knowledge without losing the quality of life of those involved in the academic process [36].

## 5. Conclusion

Our study determines that BS has an enormous prevalence in dental students, who present high levels of exhaustion and depersonalization. We also associate that these facts are commonly correlated with the person's marital status and the level of preparation (academic degree) of each individual. Among the main symptoms described by those affected by this syndrome are back (spine) pain, head and neck pain, and also TMJ.

These findings trigger an alert about the need for institutional revisions that can prioritize humanized teaching, obviously prioritizing teaching, however, without causing a loss of quality of life for the individuals involved.

## Figures and Tables

**Table 1:** Prevalence of the burnout syndrome by dimensions.

Dimensions	Low	(%)	Moderate	(%)	High	(%)
Emotional exhaustion	41	13.7	114	38.0	145	48.3^*∗*^
Depersonalization	47	15.7	113	37.7	140	46.7^*∗*^
Personal fulfillment	219	73^*∗*^	76	25.3	5	1.7

^*∗*^Presence of burnout syndrome.

**Table 2:** Descriptive analysis of burnout syndrome.

	Emotional exhaustion	Depersonalization	Personal fulfillment
*N*			
Valid	300	300	300
Losts	0	0	0
Mean	26.51	9.72	30.10
Standard error of the median	0.38	0.18	0.29
Median	26.00	9.00	31.00
Desviation	6.64	3.24	5.10

**Table 3:** Distribution of symptoms.

Symptoms	Never		Few times		Sometimes		Frequently		Always	
	*n*	(%)	*n*	(%)	*n*	(%)	*n*	(%)	*n*	(%)
Sleeping issues	31	10.3	35	11.7	12	37.3	51	17.0	71	3.7
Tension	4	1.3	12	4.0	00	33.3	67	22.3	117	9.0
Quick desitions	4	1.3	25	8.3	21	40.3	103	34.3	47	5.7
Enjoying activities	19	6.3	63	21.0	26	42.0	72	24.0	20	6.7
Headaches	18	6.0	38	12.7	96	32.0	79	26.3	69	3.0
Neck pain	18	6.0	27	9.0	94	31.3	65	21.7	96	2.0
ATM pain	107	35.7	62	20.7	71	23.7	37	12.3	23	7.7
Back and waist pain	6	2.0	21	7.0	76	25.3	82	27.3	115	38.3
Pain in the body extremities	39	13.0	66	22.0	84	28.0	47	15.7	64	21.3
Easily fatigue	17	5.7	78	26.0	23	41.0	39	13.0	43	14.3
Digestive issues	45	15.0	74	24.7	77	25.7	52	17.3	52	17.3
Lost of apetite	62	20.7	54	18.0	96	32.0	45	15.0	43	4.3
Increase of appetite	48	16.0	77	25.7	94	31.3	39	13.0	42	4.0
Nausea and vomit	130	43.3	68	22.7	61	20.3	22	7.3	19	6.3
Shaky hands	71	23.7	70	23.3	91	30.3	28	9.3	40	13.3
Respiratory issues	130	43.3	71	23.7	63	21.0	21	7.0	15	5.0

**Table 4:** Prevalence of burnout syndrome in relation to sociodemographic, academic, and symptom characteristics.

Grup	Variable	Emotional fatigue			Despersonalization			Personal fulfillment		
		*χ* ^2^	gl	*p*	*χ* ^2^	gl	*p*	*χ* ^2^	gl	*p*
Socio demographics	Age	40,916	36	0.263	32,949	36	0.614	23,366	36	0.948
	Sex	2,354	2	0.308	2,461	2	0.292	2,114	2	0.348
	Marital status	13,140	6	0.041^*∗*^	8,378	6	0.212	27,335	6	<0.001 ^*∗*^

Academics	Semester	16,594	6	0.011^*∗*^	7,295	6	0.294	5,578	6	0.472
	Shift	0.361	4	0.986	4,052	4	0.399	3,716	4	0.446
	Actuality	2,662	2	0.264	2,035	2	0.361	5,847	2	0.054
	Integral clinic	12,609	12	0.398	13,072	12	0.364	12,135	12	0.435

Symptoms	Sleeping issues	84,044	8	<0.001 ^*∗*^	44,899	8	<0.001 ^*∗*^	23,031	8	0.003^*∗*^
	Tension	105,264	8	0.00^*∗*^	25,203	8	0.001^*∗*^	22,729	8	0.004^*∗*^
	Fast response	13,125	8	0.108	7,643	8	0.469	68,761	8	<0.001 ^*∗*^
	Enjoying activities	65,554	8	<0.001 ^*∗*^	35,476	8	<0.001 ^*∗*^	45,469	8	<0.001 ^*∗*^
	Headache	68,040	8	<0.001 ^*∗*^	23,295	8	0.003^*∗*^	12,421	8	0.133
	Neck pain	67,149	8	<0.001 ^*∗*^	16,762	8	0.033^*∗*^	2,494	8	0.962
	ATM pain	32,083	8	<0.001 ^*∗*^	14,793	8	0.630	7,406	8	0.494
	Back and waist pain	39,973	8	<0.001 ^*∗*^	7,119	8	0.524	1,304	8	0.996
	Pain in the body extremities	49,847	8	<0.001 ^*∗*^	18,700	8	0.17^*∗*^	3,577	8	0.893
	Easily fatigue	67,762	8	<0.001 ^*∗*^	9,703	8	0.287	8,936	8	0.348
	Digestive issues	68,786	8	<0.001 ^*∗*^	26,223	8	<0.001 ^*∗*^	6,356	8	0.607
	Apetite issues	42,660	8	<0.001 ^*∗*^	16,731	8	0.033^*∗*^	7,510	8	0.483
	Apetite increase	13,668	8	0.091	12,005	8	0.151	17,430	8	0.026^*∗*^
	Nauseas and vomit	56,902	8	<0.001 ^*∗*^	19,815	8	0.011^*∗*^	11,765	8	0.162
	Shaky hands and shaky eyelids	68,786	8	<0.001 ^*∗*^	25,541	8	0.001^*∗*^	10,122	8	0.257
	Respiratory issues	58,662	8	<0.001 ^*∗*^	21,114	8	0.007^*∗*^	10,736	8	0.217

^*∗*^The chi-square statistic is significant at the 0.05 level.

## Data Availability

The baseline data used to support the conclusions of this study are included in the article. Detailed data used to support the conclusions of this study are available upon request from the corresponding author.
